# The effect of anti-TNF treatment on body composition and insulin resistance in patients with rheumatoid arthritis

**DOI:** 10.1007/s00296-020-04666-6

**Published:** 2020-08-10

**Authors:** I. A. M. van den Oever, M. Baniaamam, S. Simsek, H. G. Raterman, J. C. van Denderen, I. C. van Eijk, M. J. L. Peters, I. E. van der Horst-Bruinsma, Y. M. Smulders, M. T. Nurmohamed

**Affiliations:** 1 Department of Rheumatology, Amsterdam UMC, Vrije Universiteit Amsterdam, Amsterdam, The Netherlands; 2 Department of Rheumatology, Amsterdam Rheumatology and Immunology Center, Reade, Amsterdam, The Netherlands; 3 Vrije Universiteit Amsterdam, Amsterdam Cardiovascular Sciences, Amsterdam, The Netherlands; 4 Department of Internal Medicine, Northwest Clinics, Alkmaar, The Netherlands; 5 Department of Rheumatology, Northwest Clinics, Alkmaar, The Netherlands; 6 Department of Internal Medicine, Amsterdam UMC, Vrije Universiteit Amsterdam, Amsterdam, The Netherlands; 7 Amsterdam Rheumatology and Immunology Center, Amsterdam UMC, Vrije Universiteit Amsterdam, Amsterdam, The Netherlands

**Keywords:** Insulin resistance, Body composition, Rheumatoid arthritis, Osteoarthritis, Systemic inflammation

## Abstract

Given the link between systemic inflammation, body composition and insulin resistance (IR), anti-inflammatory therapy may improve IR and body composition in inflammatory joint diseases. This study assesses the IR and beta cell function in rheumatoid arthritis (RA) patients with active disease compared to osteoarthritis (OA) patients and investigates the effect of anti-TNF treatment on IR, beta cell function and body composition in RA. 28 Consecutive RA patients starting anti-TNF treatment (adalimumab), and 28 age, and sex-matched patients with OA were followed for 6 months. Exclusion criteria were use of statins, corticosteroids, and cardiovascular or endocrine co-morbidity. Pancreatic beta cell function and IR, using the homeostasis model assessment (HOMA2), and body composition, using dual-energy X-ray absorptiometry (DXA) were measured at baseline and 6 months. At baseline, IR [1.5 (1.1–1.8) vs. 0.7 (0.6–0.9), 100/%S] and beta cell function (133% vs. 102%) were significantly (*p* < 0.05) higher in RA patients with active disease as compared to OA patients. After 6 months of anti-TNF treatment, IR [1.5 (1.1–1.8) to 1.4 (1.1–1.7), *p* = 0.17] slightly improved and beta cell function [133% (115–151) to 118% (109–130), *p* <0.05] significantly improved. Improvement in IR and beta cell function was most pronounced in RA patients with highest decrease in CRP and ESR. Our observations indicate that IR and increased beta cell function are more common in RA patients with active disease. Anti-TNF reduced IR and beta cell function especially in RA patients with highest decrease in systemic inflammation and this effect was not explained by changes in body composition.

## Introduction

In healthy persons, normoglycaemia is maintained by a balanced interaction between insulin action [insulin sensitivity (IS)] and insulin secretion (pancreatic beta cell function). Insulin resistance (IR) is more prevalent in rheumatoid arthritis (RA) and is associated with endothelial dysfunction and with increased CVD [1–3]. Already in 1949, generalized muscle wasting and hyperglycaemia, both characteristics of IR, were identified in patients with RA [4]. Previous studies demonstrated that RA patients have peripheral IR that is correlated with inflammatory markers and normalizes after reduction of inflammation with glucocorticoid treatment [5–7]. Previous studies in non-diabetic RA patients found pancreatic beta cell function to be impaired [8, 9].

Increasing evidence arises that body composition, particularly body fat distribution and the quantity and characteristics of (visceral) adipose tissue, is an important factor contributing to enhanced CV risk in inflammatory diseases [3]. Cachectic obesity, defined as a loss of body cell mass without weight loss, but with concomitant increased fat mass, is a prominent feature of RA, and associated with increased circulating tumor necrosis factor (TNF) levels [10].

A clear link between obesity and chronic (low grade) inflammation was established when TNF-α, a pro-inflammatory cytokine, was found to be overexpressed in the adipose tissue of obese mice [11]. TNF-α is also overexpressed in adipose and muscle tissues of obese humans [12] and exogenous TNF-α administration leads to IR [13].

Nowadays, TNF antagonists are widely used to treat several inflammatory diseases, including RA. A recent systematic review and meta-analysis suggests that anti-TNF treatment improved IR in RA patients [14]. However, the underlying inflammatory mechanisms affecting IR in relation with body composition have not yet been fully elucidated.

To investigate the role of systemic inflammation and body composition in the development of IR, we compared RA patients, starting anti-TNF treatment, with matched osteoarthritis (OA) patients. We hypothesize that decrease in systemic inflammation and change in body composition (secondary to decrease in systemic inflammation) will positively affect IR in RA patients treated with anti-TNF. The objectives of the present investigation were (1) to investigate the body composition and IR in RA to sex and age-matched OA patients (2) to investigate correlations between IR, inflammation and body composition and (3) to study if and to what extent IR and body composition are influenced by anti-TNF treatment.

## Methods

### Study population and design

For this prospective study, 69 consecutive subjects, 36 RA and 33 OA patients were recruited from the outpatient rheumatology clinic at Reade, Center for Rehabilitation and Rheumatology, Amsterdam, the Netherlands. RA patients fulfilled the American College of Rheumatology criteria of 1987 for RA [15]. The RA patients with active disease were biological naive and were included when they were eligible for anti-TNF therapy according to the Dutch consensus statement on the initiation of anti-TNF therapy [16]. All patients started with adalimumab 40 mg every 2 weeks. The OA patients, not treated with anti-TNF, were matched for sex and age to the RA patients and they all had OA of the hands according to the American College of Rheumatology criteria of 1990 for osteoarthritis of the hand [17]. We chose OA patients as controls, because they have substantially less systemic inflammation but many similarities to RA patients in terms of life style, physical inactivity, frequent use of non-steroidal anti-inflammatory drugs (NSAIDs) and obesity. Patients, with a medical history of CVD, diabetes mellitus or hypothyroidism and/or used glucose or cholesterol-lowering medication, were excluded. Also, patients, who used systemic (oral or intramuscular) corticosteroids within a month before inclusion, were excluded from analyses. All patients were followed for 6 months and measurements were performed at *t* = 0 (before start of anti-TNF therapy and at *t* = 6 months (after start of therapy). Non-steroidal anti-inflammatory drugs (NSAIDs) were allowed to be discontinued during follow-up. When patients altered the use of anti-TNF or disease-modifying anti-inflammatory drugs (DMARDs) or started using corticosteroids, glucose, or cholesterol-lowering medication during the follow-up period, they were excluded from the follow-up analyses. This study was approved by the Ethics committee of the Slotervaart Hospital/Reade (NL19944.048.07) and prior to inclusion written informed consent from all patients was obtained.

### Patient characteristics

At baseline and after 6 months of treatment, all patients were interviewed to record details about history of co-morbidity, medication use, and disease characteristics. Special attention was paid to history of cardiovascular risk factors like hypertension, hypercholesterolemia, overweight, diabetes mellitus (DM), and smoking.

A physical examination was performed by experienced research nurses to assess blood pressure, heart rate, waist and hip circumference, length, weight, and body mass index (BMI).

Blood pressure and heart rate were measured twice (left and right) in sitting position after 5 min of rest. Hypertension was defined as a mean systolic blood pressure (SBP) ≥ 140 mmHg and/or a mean diastolic blood pressure (DBP) ≥ 90 mmHg and/or the use of antihypertensive drugs. Hypercholesterolemia was defined as total cholesterol (TC) level of ≥ 6.5 mmol/L.

Waist circumference was measured at the level of the navel, hip circumference was measured at the level of the trochanter major of the hip bone (widest circumference). The ratio of these two measurements was determined as waist–hip ratio (WHR).

Height and weight were measured using the same portable weight scale and height meter, without shoes but with clothes on. Overweight was defined as a BMI ≥ 25 kg/m^2^ and obesity as a BMI ≥ 30 kg/m^2^.

RA disease activity was assessed with the disease activity score of 28 joints (DAS28) [18] and the Health Assessment Questionnaire (HAQ) [19], a questionnaire measuring daily functioning in RA patients.

After the physical examination, an oral glucose tolerance test (OGTT) was performed. Patients were asked to drink a solution of 250 ml of water with 75 g of glucose within 5 min. Patients were not allowed to exercise or come out of the chair for the next 2 h. Two hours (plus minus 15 min) after the glucose intake blood samples were drawn to measure the glucose level.

### Blood tests

Fasting blood samples were collected to measure erythrocyte sedimentation rate (ESR), C-reactive protein (CRP), cholesterol levels (including apolipoprotein-A and B), and fasting glucose levels. All the above tests were performed the same day that blood was drawn in a single laboratory at Reade in Amsterdam. Furthermore, serum and plasma were stored at – 20 C to measure C-peptide and insulin batch-wise after all the samples were collected.

### Insulin resistance and beta cell function

The updated homeostasis model assessment (HOMA2) computer model, available from www.OCDEM.ox.ac.uk, was used to estimate insulin resistance (HOMA2-IR), and pancreatic beta cell function (HOMA2-%B) [20]. This model calculates IR and beta cell function from fasting plasma insulin and glucose concentrations and correlates well with insulin clamp method which is considered the gold standard in the assessment of insulin action in vivo [21]. This latest developed computer model has non-linear solutions as it accounts for variations in hepatic and peripheral glucose resistance and also other organs and tissues involved in glucose regulation. Pancreatic beta cell function is expressed as percentage of a normal reference population, where 100% is normal. However, HOMA2-%B measures first of all beta cell activity, not beta cell health or pathology. Therefore, the outcomes of HOMA2-%B have to be interpreted together with HOMA2-IR. Insulin resistance is expressed as 100/%insulin sensitivity (100/%S) and a normal IR is 1. The HOMA2-IR is race and age dependent of which cut-off values for abnormal insulin resistance range from 1.6 to 3.8 [22, 23]. For IR, simultaneously drawn fasting plasma glucose and insulin values were used. For beta cell function instead of insulin, C-peptide values from the same blood sample were used [24].

### Body composition

Next to the WHR and the BMI, specific body composition data, including total, gynoid (hip area) and android (abdominal area) fat percentage, lean body mass (LBM) and visceral adipose tissue (VAT) mass calculations were obtained using dual-energy X-ray absorptiometry (DXA) whole body composition. (GE Healthcare Lunar iDXA and enCORE software version 13.6) [25].

### Statistical analysis

The sample size calculation was based on the results of the of HOMA-IR measurements by Dessein et al. [26]. Based on a power analysis with alpha 0.05 and power > 85% resulted in a sample size of 25 per group.

Results are expressed as mean ± standard deviation (SD) when normally distributed, as median (interquartile range) when not normally distributed or as number and percentage. When variables were not normally distributed, the (natural) logarithms of these variables were calculated and used for analyses. Unpaired sample *t* tests were used to observe differences in baseline variables between RA and OA. For comparisons of paired continuous variables between baseline and follow-up with normal distribution paired *t* test were used. In case of non-normal distribution, we used the Wilcoxon signed-ranks test or log transformation. Pearson or Spearman correlation coefficients were determined to look for correlations between IR, IS, beta cell function, body composition measurements, and inflammatory markers. To assess the effect of change in systemic inflammation on IR and beta cell function, patients were stratified in tertiles for ΔCRP, ΔESR, and ΔDAS28. Mann–Whitney *U* tests were used to determine changes of variables after 6 months between groups. Two-sided p values less than 0.05 were considered statistically significant. All analyses were performed by IBM SPSS Statistics version 18.0.

## Results

### Study population

**Fig. 1:**
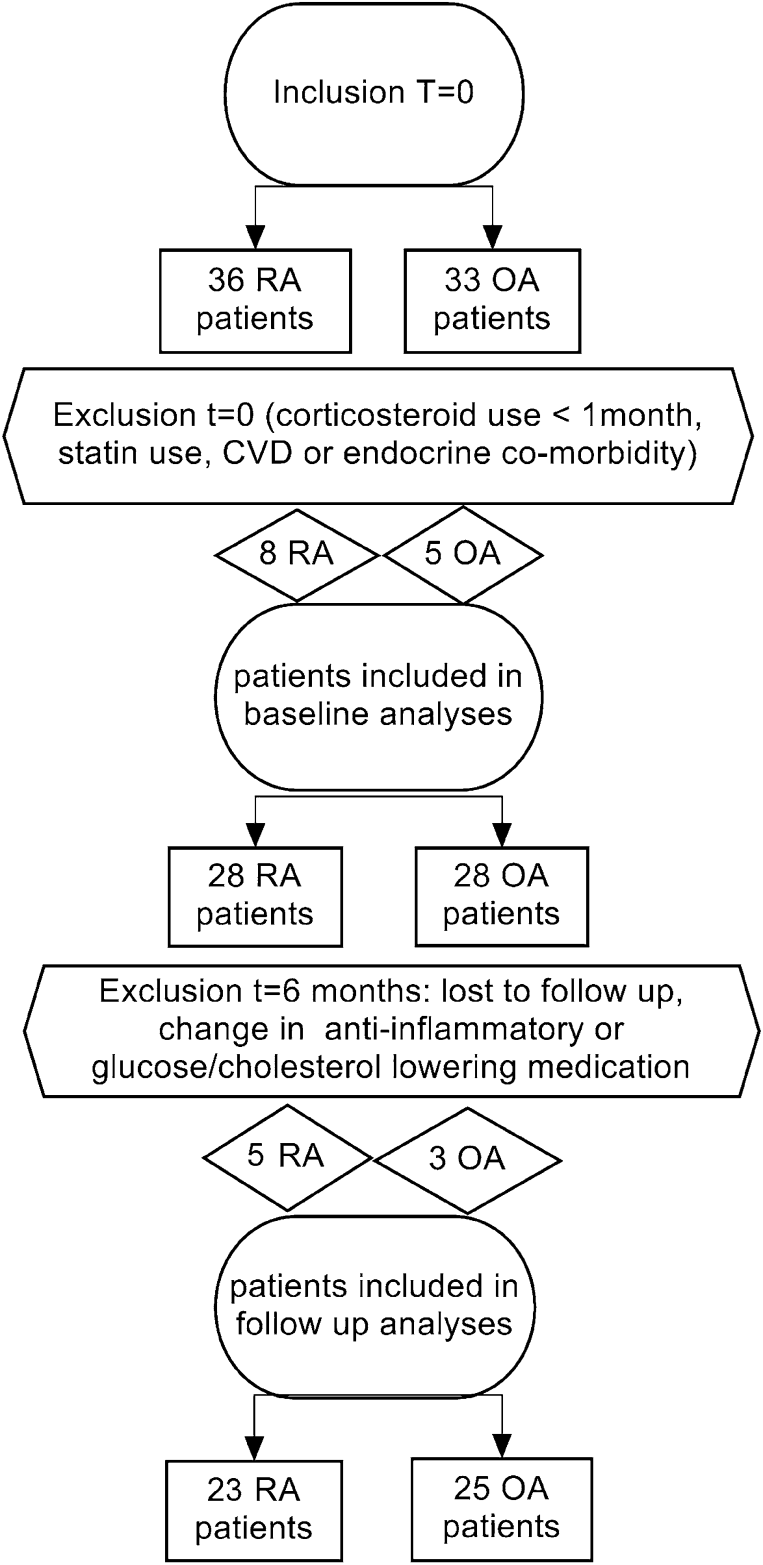
Flowchart of study inclusion

From the 69 patients, who were initially screened for study inclusion, 13 patients were excluded at baseline, mostly due to the exclusion criteria and again 9 patients were excluded at follow-up, because of discontinuation of adalimumab or loss to follow-up (Fig. 1). Baseline patient characteristics are displayed in Table 1.

**Table 1:** Baseline characteristics

	RA	OA
**Demographics**	*n* = 28	*n* = 28
Females	20 (71)	19 (68)
Age, years	53 ± 10	55 ± 11
**Cardiovascular risk factors**		
Impaired glucose tolerance^a^	3 (11)	4 (14)
Diabetes mellitus^a^	0	1 (4)
Metabolic syndrome (NCEP ATP III)	3 (11)	4 (14)
Hypertension	4 (14)	4 (14)
Antihypertensive drug use	3 (11)	4 (14)
Hypercholesterolemia	3 (10)	8 (28)
Smoking		
Current smoker	5 (18)	10 (36)
Past smoker	14 (50)	6 (21)
Never smoker	9 (32)	12 (43)
**Disease characteristics**		
Disease duration, years	4 (1–14)	1 (1–2)
Orthopedic surgery	5 (19)	8 (30)
**Anti-inflammatory medication use**		
NSAID use	14 (50)	10 (36)
DMARD use	25 (89)	0
**Rheumatoid arthritis** **specific characteristics**		
Rheumafactor positive	22 (82)	N/A
Anti-CCP positive	24 (92)	N/A
Erosive disease	15 (56)	N/A
DAS28	4.34 ± 1.35	N/A
HAQ	1.0 ± 0.6	N/A

*RA* rheumatoid arthritis, *OA* osteoarthritis, *n* number, *NSAID* non-steroidal anti-inflammatory drugs, *DMARD* disease-modifying anti-rheumatic drugs, *DAS28* disease activity score of 28 joints, *HAQ* health assessment questionnaire, *N/A* not applicable. Results are presented as mean and standard deviation (SD), median and interquartile range (IQR) or number and percentage (%)

^a ^ According to the WHO criteria for OGTT [27].

### Baseline measurements in patients with rheumatoid arthritis and osteoarthritis

**Fig. 2:**
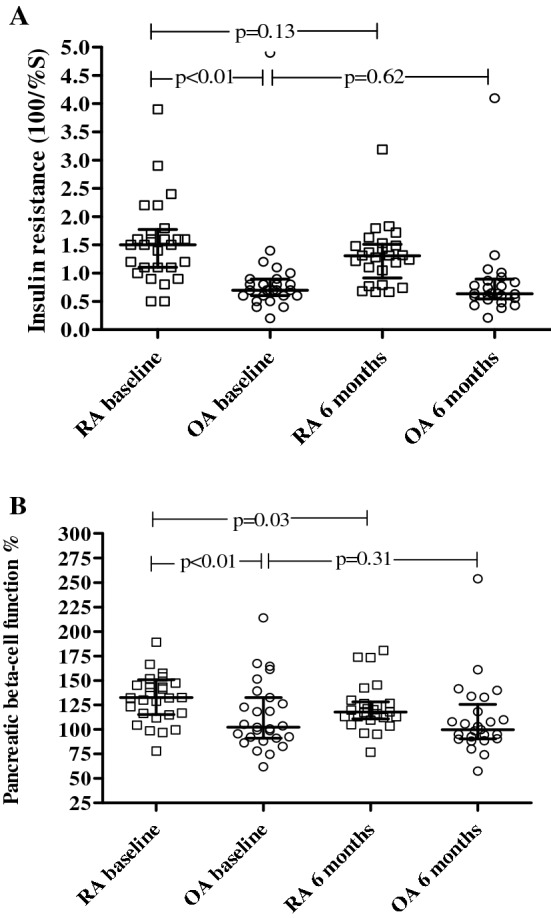
Insulin resistance and pancreatic beta cell function at baseline and after 6 months in patients with rheumatoid arthritis and osteoarthritis

**Fig. 3:**
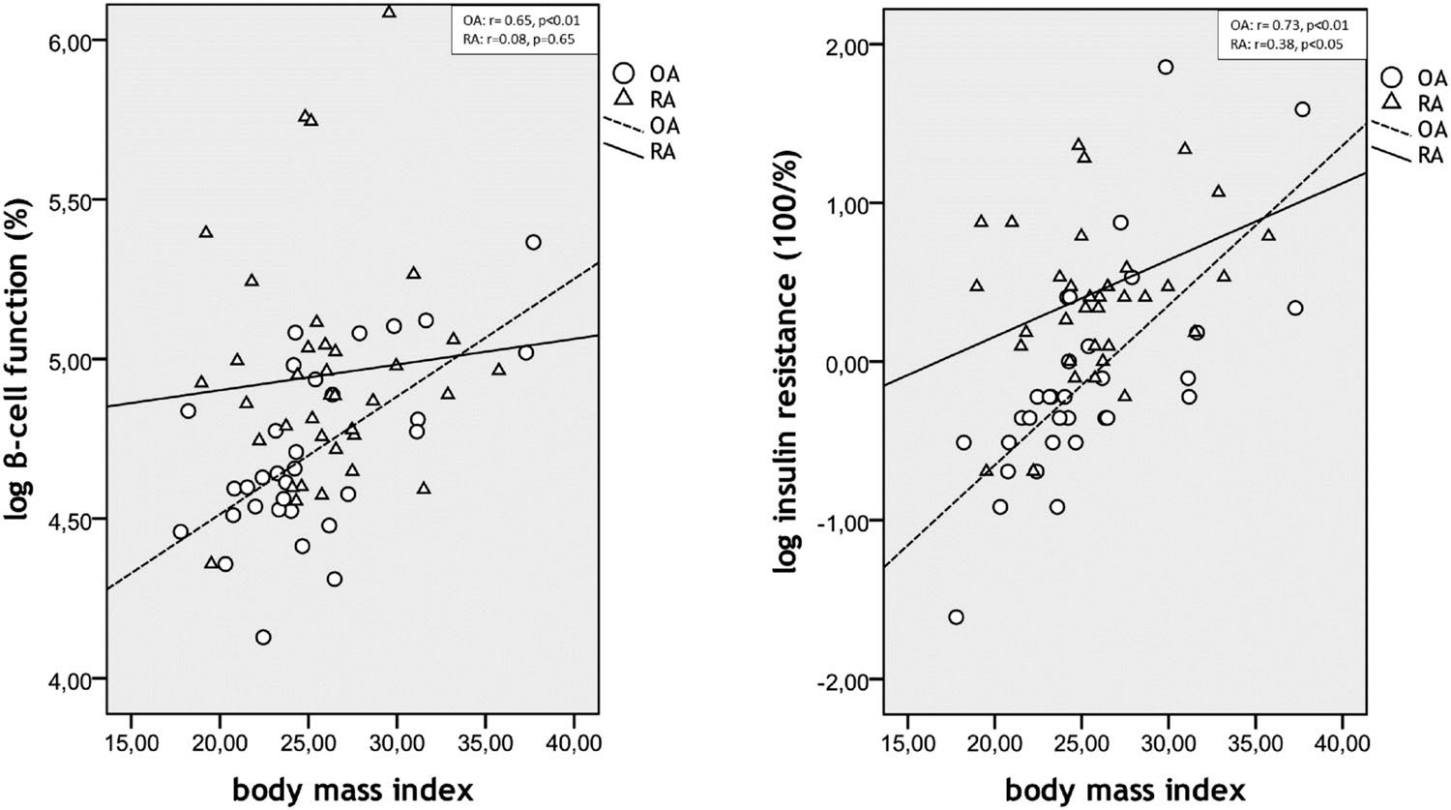
Associations between body mass index and insulin resistance and pancreatic beta cell function in rheumatoid arthritis patients (RA) and osteoarthritis patients (OA)

Table 2 presents the results of the baseline measurements in RA and OA patients. There were no significant differences in the anthropometric characteristics or blood pressure between the groups. The prevalence of metabolic syndrome determined by the NCEP ATP III guidelines [28] was 11% in the RA group and 18% in the OA group. One matched OA patient with diabetes was included erroneously but was left in according to the intention-to-treat principle. Furthermore, leaving this patient out did not affect our results. Moreover, this did not hamper our analyses of the effect of anti-TNF on the insulin resistance, as subjects did not receive any treatment and were only used as age and sex-matched controls. As expected, both ESR and CRP levels were significantly higher in the RA group. Baseline insulin levels, IR, and beta cell function were all significantly higher in RA patients compared to OA patients (Fig. 2). Fasting glucose was significantly lower in RA patients compared to OA patients (*p* < 0.05). The percentage of gynoid fat was significantly higher in RA patients compared to OA patients (*p* < 0.05). LBM and VAT mass were not significantly different between the groups. Total cholesterol, LDL-cholesterol, and apolipoprotein B were significantly lower in RA patients compared to OA patients (Fig. 3).

**Table 2:** Results of measurement changes after 6 months comparing rheumatoid arthritis patients with anti-TNF treatment to osteoarthritis patients without anti-TNF treatment

	RA group *n* = 28 *t* = 0	RA group *n* = 23 *t* = 6 months	*p* (difference paired values)	OA group *n* = 28 *t* = 0	OA group *n* = 25 *t* = 6 months	*p* (difference paired values)	*p* (difference baseline values)
Physical examination							
Systolic blood pressure, mmHg	126 ± 17	123 ± 16	0.19	122 ± 12	118 ± 11	0.14	0.26
Diastolic blood pressure, mmHg	76 ± 9	72 ± 8	< 0.01*	72 ± 9	70 ± 8	0.21	0.18
Waist–hip ratio	0.88 ± 0.07	0.87 ± 0.07	0.63	0.87 ± 0.07	0.87 ± 0.076	0.69	0.40
Body mass index, kg/m^2^	26.2 ± 4.0	25.7 ± 3.5	0.80	25.3 ± 4.9	25.7 ± 5.1	0.12	0.41
DAS28	4.34 ± 1.35	2.76 ± 0.94	< 0.001*	–	–	–	–
Health assessment questionnaire	1.1 (0.4–1.5)	0.5 (0–1.3)	0.001*	–	–	–	–
Inflammatory markers							
ESR, mm/hour	17 (11–32)	14 (6–17)	< 0.01*	7 (3–11)	5 (4–9)	0.25	< 0.001*
CRP, mg/l	5 (3–9)	2 (2–4)	< 0.01*	2 (1–3)	2 (1–3)	0.62	< 0.01*
Glucose and insulin measurements							
Fasting glucose, mmol/l	4.9 (4.7–5.1)	4.9 (4.6–5.4)	0.32	5.2 (4.8–5.4)	5.2 (4.9–5.5)	0.94	< 0.05*
Glucose 2 h after 75 g glucose, mmol/l	5.6 (4.5–6.5)	5.6 (4.2–6.8)	0.87	5.0 (4.2–5.7)	4.7 (4.2–5.8)	0.85	0.14
C-peptide nmol/l	0.71 (0.58–0.80)	0.64 (0.51–0.76)	< 0.05*	0.52 (0.46–0.71)	0.56 (0.44–0.68)	0.55	0.10
Insulin, µU/ml	12.0 (8.4–13.7)	9.8 (6.2–11.5)	0.14	5.7 (4.3–7.0)	4.9 (4.17–6.82)	0.71	< 0.01*
Insulin resistance (100/%S)	1.5 (1.1–1.8)	1.4 (1.1–1.7)	0.17	0.7 (0.6–0.9)	0.64 (0.55–0.9)	0.75	< 0.01*
Beta cell function, % (Cpep)	133 (115–151)	118 (109–130)	< 0.05*	102 (91–133)	100 (90–125)	0.3	< 0.01*
Body composition whole body DXA							
Fat percentage whole body	37 ± 11	37 ± 8	0.52	35 (29–43)	35 (27–45)	0.18	0.77
Gynoid fat percentage	43 ± 11	41 ± 10	0.01*	37 ± 10	37 ± 10	0.75	< 0.05*
Android fat percentage	43 ± 12	41 ± 10	0.20	38 (31–49)	40 (29–49)	0.13	0.13
Visceral adipose tissue mass, grams	888 (492–1436)	870 (473–1350)	0.93	722 (435–1098)	785 (455–1249)	< 0.05*	0.68
Lean body mass whole body, kilograms	46.1 ± 7.6	46.7 ± 7.7	0.25	46.1 ± 9.7	46.5 ± 9.6	0.92	0.99
Lipid profile							
Total cholesterol, mmol/l	5.02 ± 0.95	5.47 ± 1.00	< 0.01*	5.88 ± 1.01	5.70 ± 0.99	< 0.05*	< 0.01*
Triglycerides, mmol/l	1.13 ± 0.50	1.20 ± 0.56	0.49	1.09 (0.94–1.54)	1.17 (0.89–1.55)	0.47	0.12
LDL-cholesterol, mmol/l	3.04 ± 0.87	3.33 ± 0.92	< 0.05*	3.76 ± 0.96	3.64 ± 0.90	< 0.05*	< 0.01*
HDL-cholesterol, mmol/l	1.48 ± 0.34	1.66 ± 0.54	< 0.05*	1.53 ± 0.41	1.49 ± 0.34	0.60	0.59
Total cholesterol/HDL ratio	3.69 ± 1.17	3.58 ± 1.18	0.41	4.10 ± 1.24	4.02 ± 1.13	0.26	0.17
Apolipoprotein A, g/l	1.67 ± 0.29	1.83 ± 0.48	< 0.05*	1.73 ± 0.28	1.68 ± 0.25	0.17	0.47
Apolipoprotein B, g/l	0.92 ± 0.27	0.99 ± 0.26	0.084	1.07 ± 0.24	1.07 ± 0.26	1.0	< 0.05*

*RA* rheumatoid arthritis, *OA* osteoarthritis, *n* number, *NCEP ATP III* National Cholesterol Education Program Adult Treatment Panel III (US 2001), *DXA* dual-energy X-ray absorptiometry

Results are presented as mean and standard deviation (± SD), median and interquartile range (IQR) or number percentage

*Significance level of* p* < 0.05

### Inflammation, insulin resistance, and beta cell function

**Fig. 4:**
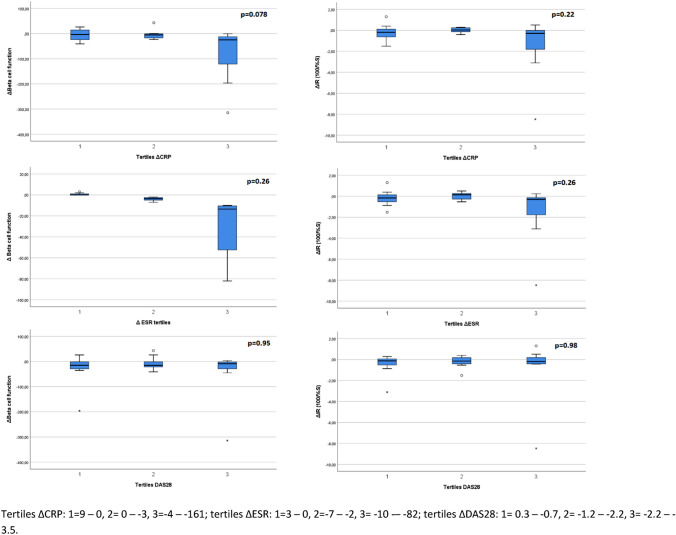
Change in insulin resistance and pancreatic beta cell function in RA patients after 6 months anti-TNF treatment categorized in tertiles for delta inflammatory parameters

To analyse the association between change in systemic inflammation and change in IR and beta cell function, patients were stratified in equal tertiles of delta inflammatory parameters, i.e. ΔCRP, ΔESR, and ΔDAS28. Results are displayed in Fig. 4.

### Correlations between insulin resistance, beta cell function, and body composition data per group

In the RA group, except for a correlation between BMI and IR (*r* = 0.38, *p* < 0.05) (see Fig. 3), no significant correlations were found between IR or beta cell function and body composition (data not shown).

In the OA group, IR and beta cell function were correlated (*p* < 0.01) with BMI (*r* = 0.73, *r* = 0.65 respectively, Fig. 3), whole body fat percentage (*r* = 0.65, *r* = 0.60 respectively), android fat percentage (*r* = 0.72, *r* = 0.56, respectively) and (*p* < 0.05) gynoid fat percentage (*r* = 0.39, *r* = 0.48, respectively). IR but not beta cell function was also significantly correlated with VAT mass (*r* = − 0.66, *p* < 0.01, *r* = 0.31, *p* = 0.14, respectively) and LBM (*r* = 0.41, *p* = 0.04, *r* = 0.11, *p* = 0.58, respectively).

### The effect of anti-TNF treatment

The results of the comparisons between OA and RA patients after 6 months anti-TNF treatment are displayed in Fig. 2 and Table 2. Eventually, five RA patients and three OA patients were excluded from follow-up due to lost to follow-up, change in anti-inflammatory treatment, and change in glucose/cholesterol-lowering medication. Both inflammatory markers ESR and CRP decreased significantly in the RA group compared to the OA group. Also DAS28 and HAQ decreased significantly in RA. In contrast to the OA patients, in the RA group, beta cell function and gynoid fat percentage decreased significantly (*p* < 0.05 and *p* = 0.01). Total cholesterol, LDL, apolipoprotein A, and also HDL increased significantly after 6 months of treatment in the RA group compared to the OA group (*p* < 0.01). However, total cholesterol/HDL ratio did not change at follow-up.

## Discussion

Insulin resistance (IR) is increased in rheumatoid arthritis (RA) and it is assumed that IR, systemic inflammation, and body composition are interrelated. We hypothesized that a decrease of systemic inflammation and change in body composition would favourably affect IR in RA patients. The present study showed: (1) IR and beta cell function were significantly higher in RA than in OA; (2) no significant correlations were found between IR or beta cell function and body composition in RA patients; (3) pancreatic beta cell function and IR improved after anti-TNF treatment in RA patients with highest decrease of CRP and ESR, albeit that this did not reach statistical significance.

This latter effect might be due to a decrease in hyper-metabolism and resting energy expenditure that comes along with inflammation and active disease [29, 30]. However, there was no clinical relevant alteration of the overall body composition.

Together with the inflammatory markers, IR and beta cell function were significantly higher in RA than in OA. Overall, in the OA group, IR and beta cell function were in the normal range, whereas in the RA group, IR and beta cell function were both raised. Inflammation is considered crucial in the pathogenesis of IR. Therefore, these observed differences were expected as RA is a high-grade inflammatory disease compared to OA which is a low-grade inflammatory disease [2]. When we investigated the effect of systemic inflammation in more detail by dividing the RA patients in tertiles according to change in CRP, ESR and DAS28 levels, we found that IR and especially beta cell function improved most after anti-TNF treatment in the RA patients with the highest decrease of CRP and ESR. This suggests that reducing inflammation also improves IR and beta cell function, whether or not this is anti-TNF specific or caused by inflammation reduction [31]. As we found beta cell function equally raised compared to IR and also found no differences between RA and OA in impaired glucose tolerance overall, it’s unlikely that beta cell function was significantly impaired in our group of RA patients as was found by Ferraz-Amaro et al. [8].

In OA patients there were clear correlations between IR, beta cell function and BMI and especially android fat percentages, as expected, while in RA only BMI was modestly correlated with IR. Total fat percentage and BMI were slightly higher in RA, but except for gynoid fat percentage, which was significantly higher in RA compared to OA, no significant differences were found in android fat percentage and VAT or LBM, which we had expected. This is to some extent in contrast to the existing literature where increases of BMI and body fat after anti-TNF have been described [32]. Generally, increases of BMI and body fat and BMI were observed after more than a year treatment. Therefore, the 6 months anti-TNF treatment in our study might have been be too short to detect changes in body composition; particularly as in other studies also, no changes in BMI and/or body composition were observed after 6 months or shorter duration of anti-TNF treatment [32]. Altogether, these data show that inflammation rather than an altered body composition explains the higher IR and beta cell function in RA; however, further research to discover the exact pathophysiologic mechanism is still needed. Theoretically, a direct effect of anti-TNF on the insulin pathway and fat metabolism cannot be ruled out. However, as more studies demonstrated a correlation between systemic inflammation and insulin resistance, it is plausible to assume the impact of anti-TNF is based on the anti-inflammatory effect. This is underscored by several studies where the effect of other anti-inflammatory anti-rheumatic drugs therapy on the insulin resistance in RA patients was investigated [33, 34]. No significant differences in total fat percentage and VAT mass between RA and OA patients were found. This might be explained by the fact that OA is associated with obesity and physical inactivity leading to an altered body composition in comparison to the general population [35]. Furthermore, OA is a disease which has many similarities with RA, like for instance chronic pain and joint deformity that both could result in reduced physical activity. Our findings suggest that reduced physical activity and inflammation play an important role in the body composition of both groups, but presumably in different ways and gradations.

Strength of this study was that factors that could have influenced the results, i.e. use of corticosteroids, statin use, a history of CVD or metabolic diseases were excluded. Moreover, changes in medication use during the study period were prohibited. With OA patients as controls, the effect of anti-TNF treatment could be better evaluated, as time can also influence results. Our study has its limitations. It may be argued that the study duration was very short to capture the metabolic effects of anti-TNF on the insulin action and body composition. Nonetheless, as several studies did observe similar effects after comparable treatment period [14, 32], this did not hamper the assessment of the relation between systemic inflammation and insulin resistance.

In conclusion, this study showed that IR and beta cell function are increased in RA patients compared to OA patients. Anti-TNF treatment improved IR and beta cell function in RA patients with highest decrease of CRP and ESR which was not explained by change in body composition. The data suggest that this is caused by lowering of inflammatory activity in general; however, a direct effect by blocking TNF cannot be excluded in this study and needs further investigation. Nevertheless, our data indicate that reducing systemic inflammation and disease activity reduces IR and beta cell function which ultimately might lessen the CV disease burden.

## Data Availability

The datasets generated during and/or analysed during the current study are available from the corresponding author on reasonable request.
